# Inequity in access to digital public primary healthcare in Sweden: a cross-sectional study of the effects of urbanicity and socioeconomic status on utilization

**DOI:** 10.1186/s12939-024-02159-7

**Published:** 2024-04-15

**Authors:** Jon Eriksson, Susanna Calling, Ulf Jakobsson, Moa Wolff, Beata Borgström Bolmsjö, Veronica Milos Nymberg

**Affiliations:** 1 Center for Primary Health Care Research, Department of Clinical Sciences Malmö, Lund University, Malmö, Sweden; 2 Primary care Skåne, Region Skåne, Kristianstad, Sweden; 3 Center for Primary Health Care Research, Clinical Research Centre, 202 13 Malmö, Box 50332, Sweden

**Keywords:** Digital healthcare, Health equity, Healthcare utilization, Urbanicity, Socioeconomic status

## Abstract

**Background:**

Social and geographical inequity in access to primary healthcare is an ongoing concern in Sweden. Digital care can potentially decrease geographical inequity. This study aimed to evaluate how urbanicity affects the utilization of a public digital primary healthcare service - PHC Online.

**Methods:**

We performed an ecological cross-sectional study of 4,482 PHC Online visits grouped by 83 public primary healthcare centers. Multiple linear regression analysis was performed with PHC Online visits per 1,000 registered patients as the dependent variable and urbanicity (municipalities grouped by number of inhabitants), socioeconomic status (Care Need Index), and morbidity (Adjusted Clinical Groups) per primary healthcare center as independent variables.

**Results:**

Utilization of PHC Online was more common among those of a younger age (median 32 years) and among women (65%). Urbanicity did not affect utilization. Lower socioeconomic status and higher morbidity had negative effects on utilization (B -3.289, *p* = 0.001, B -7.728, *p* = 0.045).

**Conclusions:**

Geographical differences based on urbanicity do not seem to affect the utilization of PHC Online. Further studies are needed to clarify a possible association to geographical barriers in access to primary healthcare, specifically accounting for factors associated with urbanicity and distance to physical clinics, and how age and sex affect such an association. Lower utilization of PHC Online in low socioeconomic status and high morbidity populations raises questions on the effect of digital primary care on equitable access to primary healthcare.

## Background

Despite overall levels of good health across the population in Sweden, health disparities according to social conditions such as age, education and place of residence are present [1]. Limited access to primary healthcare (PHC) (ability to utilize services according to conditions) can serve as a barrier to obtaining and maintaining health [2]. Therefore, equity in health requires equity in access to PHC. Marketization reforms during recent decades have contributed to an increase in already existing geographical inequity in access to PHC, with an increase of primary healthcare centers (PHCC) in urban and socioeconomically advantaged areas [3]. Simultaneously, Sweden has had a rapid growth of direct-to-consumer telemedicine services (digital PHC) [4]. While digital PHC has the potential to decrease geographical inequity in access to PHC, utilization has so far been more frequent among groups with fewer geographical barriers to access PHC (residency in urban areas) [4, 5]. Similar results have been found internationally [6–8]. Digital PHC also seems to increase existing health disparities according to social conditions with utilization being more frequent among groups with expected lower healthcare needs (young age, high income, high education, and indigenous country of birth) [4, 5]. Previous studies have argued that skills and attitudes associated with the effective use of digital systems (digital health literacy) are less frequent in disadvantaged social groups and older age groups [9, 10]. However, not only patients’ behaviors but also healthcare providers’ behaviors might affect this development. Since the introduction of free establishment and competition in Swedish PHC, resource allocation has become more dependent on provider location, patient choice, and demand, and less on healthcare need, according to a scoping review [3]. To attract more patients, digital healthcare providers might be more prone to target patient groups in urban areas with high digital health literacy and, in the process, increase geographical and social inequity in access to PHC and health.

Digital PHC in Sweden was first implemented by private companies but recently several publicly run services have been established, one such being Primary Healthcare Scania Online (PHC Online) by Region Skåne. Swedish law states that healthcare shall be provided on equal terms and priority shall be based on need [11]. Furthermore, Sweden has set up goals for the digital development of healthcare to be non-discriminatory and based on different group’s needs [12].

Inequity in the utilization of digital PHC according to social conditions is well established [4–8, 10]. The current development in Sweden may be encouraged by competition rather than a genuine need for healthcare, potentially leading to increased inequity in health and failure to achieve established policy goals. With other social disadvantages negatively affecting the utilization, the ability to decrease geographical inequity in access is an important factor in fulfilling digital PHCs’ ultimate goal of increasing (equitable) access to PHC. Therefore, the aim of this study was to evaluate how geographical differences in urbanicity affect a population’s utilization of PHC Online.

## Methods

### Setting

Region Skåne is the southernmost and third most populated of the 21 self-governing regions in Sweden with around 1.4 million inhabitants [13] and 33 municipalities. The mean life expectancy of the population of Region Skåne is approximate to Sweden as a whole (84.5 years for women and 81 years for men) [14], the mean age of the population is the third lowest of the regions in Sweden (41.3 years) [15] and the amount of foreign-born people is larger than for Sweden as a whole (23.5%) [16]. A total of 156 publicly funded PHCCs (either publicly or privately operated) existed within Region Skåne in the study year 2021 and these constituted the absolute majority of PHC in the region. PHC Online was launched in 2019 as a digital PHC service and, apart from this, the population of Region Skåne also had access to several privately run digital PHC services. Though open to all, PHC Online specifically targeted inhabitants registered at publicly operated PHCCs in Region Skåne, via advertisement and reference. Inhabitants registered at privately operated PHCCs, or in other regions, in most cases had access to alternative digital services. Healthcare professionals operated the service between 8am − 5pm on weekdays and between 10am − 3pm on weekends but patients could access, and initiate a visit, at any time. PHC Online specifically targeted mild conditions deemed suitable for digital healthcare (such as allergies or skin rashes) but all patients seeking care got a medical assessment. Interaction with healthcare professionals was mainly done via synchronous or asynchronous chat, with the possibility to convert the communication into video visits if needed.

### Study design and population

Data on all completed visits to PHC Online between February - December 2021, patients’ registered PHCC, diagnoses according to International Statistical Classification of Diseases and Related Health Problems (ICD-10), age, sex, and healthcare profession category were acquired from Region Skåne’s Health Care Databases (RSVD). Data on existing PHCCs, number of registered patients, location according to municipality and mode of operation (public or private) in 2021 were acquired from Region Skåne. Data on Care Need Index (CNI), Adjusted Clinical Groups (ACG) (see further explanation below), and total number of visits for each PHCC in 2021 were acquired from Region Skåne’s public website. Number of inhabitants and mean age of population for each municipality in December 2021 was acquired from the public website of Statistics Sweden [13, 17]. Inclusion criteria were: visits to physicians completed by patients registered to PHCCs in Region Skåne. Exclusion criteria were: privately operated PHCCs and PHCCs opening or closing during the study period. Visits to nurses were excluded since a national telephone service facilitating medical assessment and advice by nurses already exists in Sweden. Privately operated PHCCs were excluded due to three reasons: (1) PHC Online specifically targeted patients registered to public PHCCs; (2) patients registered to privately operated PHCCs had access to alternative digital services, and (3) the frequency of completed visits to PHC Online by patients registered to privately operated PHCCs was relatively low. Data were grouped to each PHCC resulting in a cross-sectional ecological study of completed visits to physicians from patients registered to publicly operated PHCCs.

### Variables

#### Outcome

For each PHCC the number of PHC Online visits per 1,000 registered patients was calculated resulting in the continuous variable “PHC Online visits”. We used mean, minimum, and maximum to describe the normally distributed variable. Within each category of urbanicity (see description below), we used median, minimum, and maximum to describe the variable since it was not normally distributed within these categories.

#### Age

Frequencies of PHC Online visits per age (in years) were analyzed and presented as median, minimum, maximum, and percentiles. Duplicate visits completed by the same patient were included. For urbanicity groups, the mean age and standard deviation of the municipality populations was calculated for each category.

#### Sex

Frequencies of PHC Online visits were analyzed using the percentage of PHC Online visits, including duplicates, per category.

#### Diagnosis

Diagnosis was grouped according to ICD-10 chapters. Within each group, the three most common diagnoses were determined except for “Diseases of the genitourinary system” since 80% of this group was represented by a single diagnosis.

#### Geographical differences (urbanicity)

Municipalities were classified into three groups using population to determine urbanicity: (A) large cities (population > 100,000), (B) medium-sized towns (population 20,000-100,000) and (C) smaller towns and rural municipalities (population < 20,000) resulting in the “urbanicity” category variable. One exception was made for a municipality adjacent to a large city that had shared infrastructure and no obvious barriers between them. This municipality was classified as B instead of C. Utilizing municipality data, we employed the total number, median, minimum, and maximum to describe population differences between categories of urbanicity.

#### Socioeconomic status (CNI)

CNI, a continuous variable, was used as a proxy for socioeconomic status (SES) for each PHCC’s population. CNI is an index used for expected healthcare resource needs within a population, based on seven socioeconomic factors: number of people that are unemployed, are below the age of five years, foreign-born (East Europe, Asia, Africa or South America), are single parents with children below the age of 18 years, are single and above the age of 65 years, have moved within the last year, and have low education [18]. A higher CNI indicates a lower SES and a higher healthcare need [19]. Using PHCC data, we used median, minimum, and maximum to describe differences in CNI between categories of urbanicity.

#### Morbidity (ACG)

ACG, a continuous variable, was used as a proxy for morbidity for each PHCC’s population. ACG is an index used for expected healthcare resource needs within a population based on factors related to morbidity: diagnoses per patient in relationship to duration, severity of disease, diagnostic certainty, cause of illness, requirements of specialist care, and average resource usage in PHC [20]. A higher ACG indicates a higher morbidity rate and a higher healthcare need [21, 22]. Using PHCC data, we used mean and standard deviation to describe differences in ACG between categories of urbanicity.

### Data analysis

All statistical analyses were done in SPSS version 29.0. To minimize the risk for multicollinearity, correlations between variables were tested using Pearsons’s test for normally distributed variables and Spearman’s test for not normally distributed variables. No variable correlated > 0.5 and hence all were included in the regression analyses. Dummy variables for the different categories of urbanicity were created and coded “1” and “0”. Multiple linear regressions were conducted with PHC Online visits as the dependent variable and urbanicity (reference category “large cities”), CNI, and ACG as independent variables. Residuals in the regression models were tested and found normally distributed. Variance Inflation Factor (VIF) < 4 and Tolerance > 0.25 were used to assess multicollinearity in linear regression.

## Results

### Descriptives

**Fig. 1:**
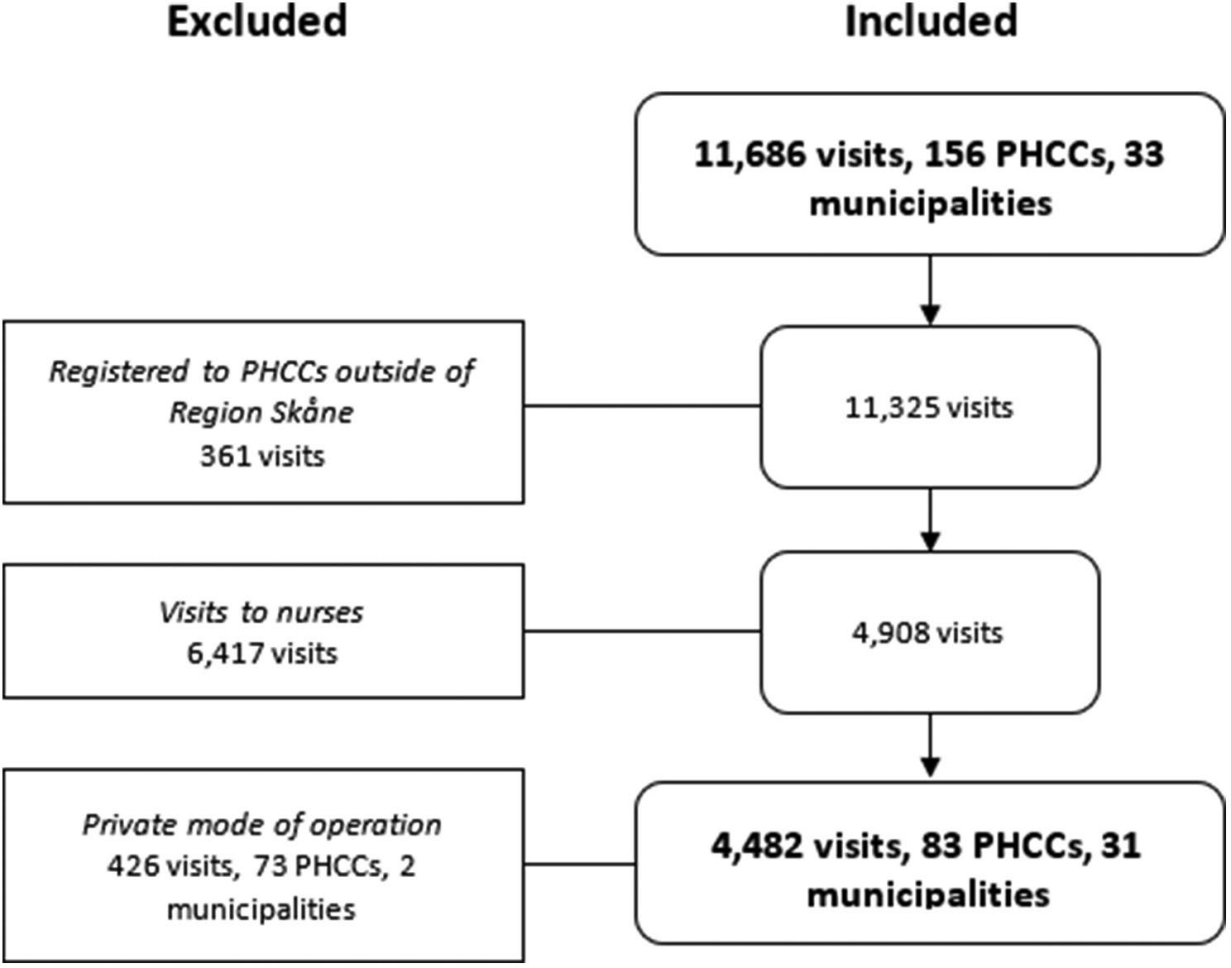
Included visits, PHCCs, and municipalities according to inclusion and exclusion criteria. *PHCC (primary healthcare center)*

During the study period, a total of 11,686 visits were completed to PHC Online. 361 visits were excluded from patients registered to PHCCs outside of Region Skåne and 6,417 visits to nurses were excluded. Of the 156 PHCCs, 73 were excluded due to being privately operated, which left 83 PHCCs. All of these existed during the entire study period. Patients registered to these 83 PHCCs completed 4,482 visits to physicians and the PHCCs were located in 31 of the 33 municipalities (Fig. 1). These represent the final study population.

**Fig. 2:**
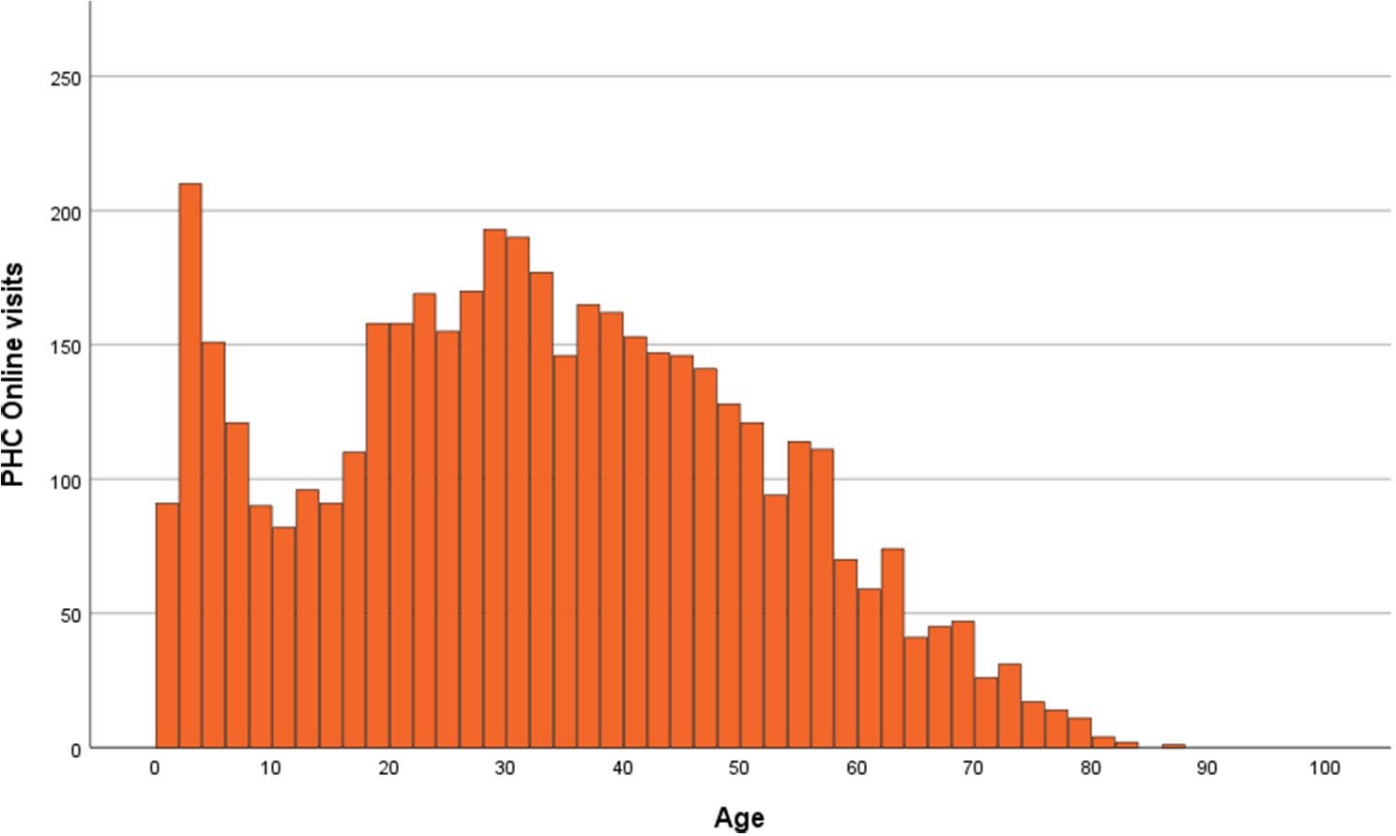
Distribution of number of PHC online visits per age in years. *PHC Online (Primary Healthcare Scania Online)*

3,653 patients completed a total of 4,482 visits to PHC Online during the studied period. The age distribution for visits was wide but younger age was more common with a minimum of one year, a maximum of 86 years, a median of 32 years, and the 90th percentile at 57 years (Fig. 2).

**Fig. 3:**
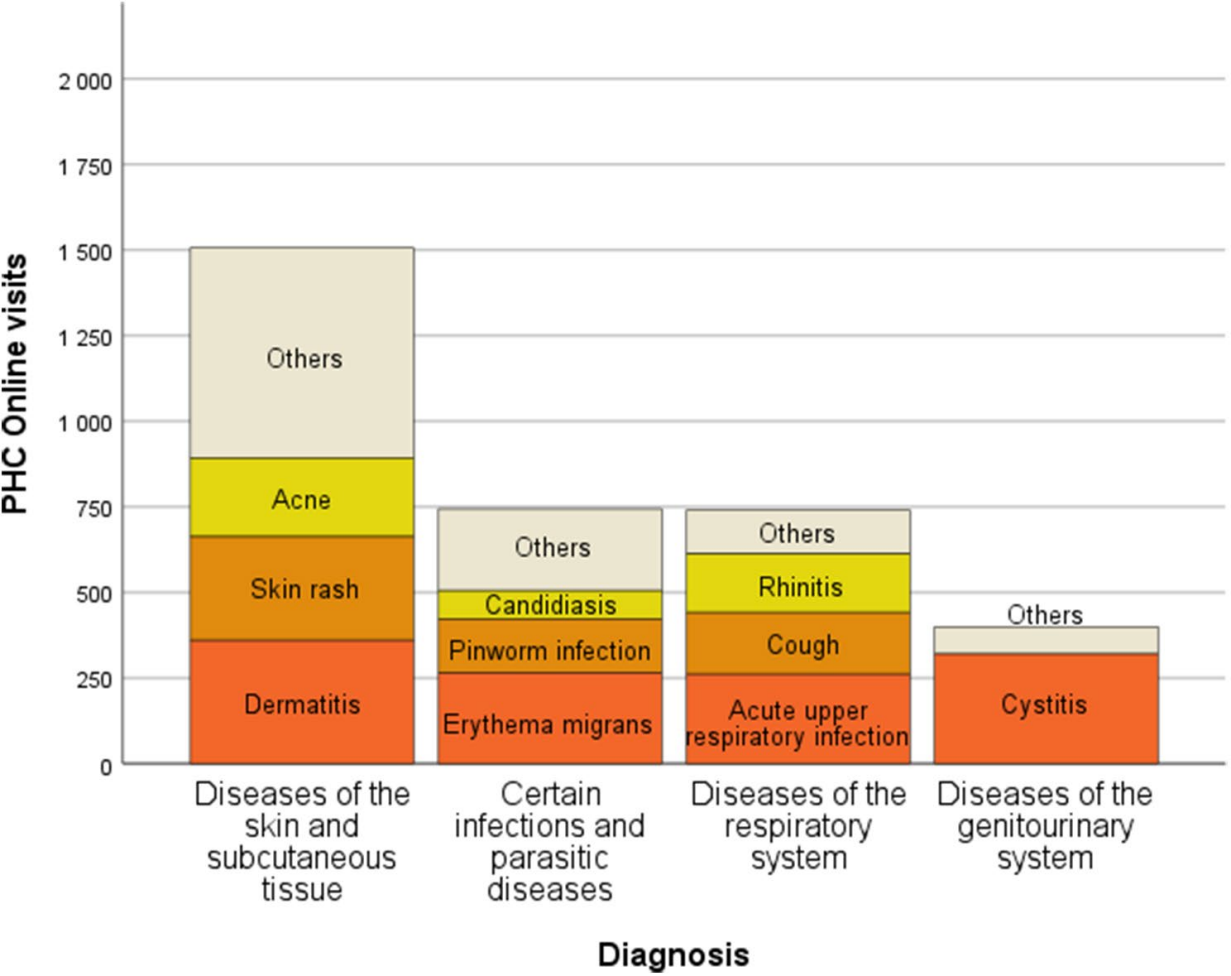
Distribution of the most common diagnoses in number of visits per diagnostic group and most common diagnoses within groups. *PHC Online (Primary Healthcare Scania Online)*

Women completed 65% of the visits and men 35%. The most common diagnoses were diseases of the skin (33.6%), certain infections (16.6%), diseases of the respiratory system (16.5%), and diseases of the genitourinary system (8.9%) of which 80% were cystitis (Fig. 3). Together these groups represented 75.7% of the visits.

**Fig. 4:**
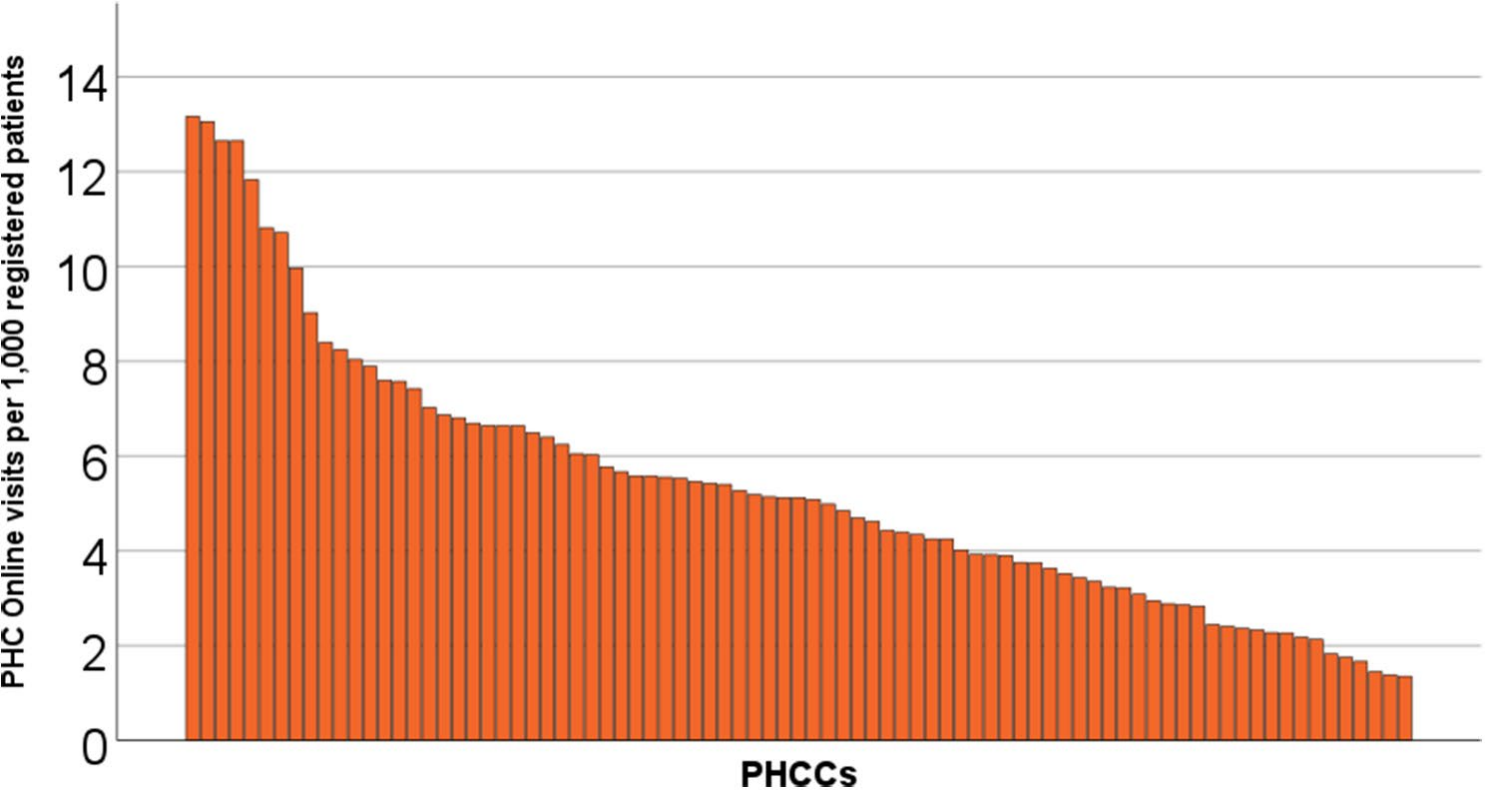
PHC online visits per 1,000 registered patients per PHCC. *PHCC (primary healthcare center), PHC Online (Primary Healthcare Scania Online)*

The number of PHC Online visits for the 83 included PHCCs varied (had a range) between 1.35 visits/1,000 registered patients and 13.17 visits/1,000 registered patients (mean 5.37) (Fig. 4).

There were gradients with higher urbanicity showing lower age, higher number of PHC Online visits and lower morbidity (lower ACG). SES (CNI) did not show a gradient between categories of urbanicity (Table 1).

**Table 1:** Population number (thousands), population age (years), PHC Online visits (per 1,000 registered patients), CNI, and ACG per urbanicity (A) large cities, (B) medium-sized towns, and (C) smaller towns and rural municipalities

Urbanicity	A. Large cities	B. Medium-sized towns	C. Smaller towns and rural municipalities
**Municipalities** *(n)*	*(3)*	*(14)*	*(14)*
**Population number**, total	629	533	209
**Population number**, median *(min-max)*	150 *(127–352)*	34 *(20–87)*	16 *(8–20)*
**Population age**, mean *(SD)*	39 *(1)*	42 *(2)*	43 *(2)*
**PHCCs** *(n)*	*(36)*	*(31)*	*(16)*
**CNI**, median *(min-max)*	1.09 *(0.63-1.93)*	0.92 *(0.63-2.03)*	0.97 *(0.81-1.33)*
**ACG**, mean *(SD)*	0.96 *(0.09)*	1 *(0.07)*	1.04 *(0.07)*
**PHC Online visits**, median *(min-max)*	5.29 *(1.35–13.17)*	4.85 *(1.38–13.06)*	4.64 *(1.75–9.02)*

PHC Online (Primary Healthcare Scania Online), CNI (Care Need Index), ACG (Adjusted Clinical Groups), SD (Standard deviation)

### Linear regression

Urbanicity had no statistically significant effect on the utilization of PHC Online. High SES (low CNI) and low morbidity (low ACG) both had statistically significant effects on the utilization of PHC Online which increased when adjusting for urbanicity and CNI and ACG respectively (Table 2).

**Table 2:** Multiple linear regression with the outcome PHC Online visits per 1,000 registered patients using independent variables urbanicity (reference category: Large cities), CNI, and ACG.

	Unadjusted	Adjusted		
	B *(p-value)*	B *(p-value)*	**Tolerance**	**VIF**
**Urbanicity**				
Medium-sized towns	0.171 *(0.793)*	− 0.479 *(0.499)*	0.732	1.367
Smaller towns and rural municipalities	− 0.750 *(0.345)*	− 0.899 *(0.304)*	0.726	1.377
**CNI**	-2.562 *(0.008)*	-3.289 *(0.001)*	0.869	1.150
**ACG**	-6.681 *(0.075)*	-7.728 *(0.045)*	0.863	1.159

VIF (Variance Inflation Factor), PHC Online (Primary Healthcare Scania Online), CNI (Care Need Index), ACG (Adjusted Clinical Groups)

*n = 83

**R²: 0.164

## Discussion

### Main findings

Our study shows that utilization of PHC Online was more common among those of a younger age and women and that the most common diagnoses were mild conditions (such as dermatitis and cystitis). There was a difference between PHCCs in PHC Online visits, but urbanicity did not affect the utilization of PHC Online. CNI and ACG had significant negative effects on utilization of PHC Online, which shows that utilization was more common among low morbidity and high SES populations. This indicates a social inequity in access to PHC Online and questions access being aligned with the need for healthcare.

Possible explanations for the difference in utilization between sexes are lower healthcare-seeking behavior among men and some targeted conditions being more frequent among women (e.g. cystitis). Since PHC Online specifically targets mild conditions, this study’s findings on most common diagnoses are not comparable to a physical PHCC’s diagnose distribution. All visits included in this study were triaged by a nurse and the pattern of mild conditions being the most common does therefore not represent a potentially unnecessary use of healthcare resources. Targeting mild conditions could also explain low morbidity being associated with the utilization of digital PHC. It remains uncertain to what extent this further affects the utilization of physical PHC. A decrease in utilization of physical PHC by low-morbidity populations might increase access for high-morbidity populations. But as medical staff working with PHC online were also employed at PHCCs, increasing utilization of digital visits could hypothetically contribute to a displacement of care, where populations with higher ACG and CNI and in need of physical examinations get less access to PHC. As we did not have access to data on staff and resource utilization, we were not able to draw any conclusions about this assumption. The effect of PHC Online on access to physical care in populations with high care needs (as a proxy for equitable resource allocation) remains to be investigated by future studies.

### Comparison with other studies

A previous study of a Swedish population showed utilization of digital PHC being more common among those of a younger age and women [4] and a previous study of an American population showed similar results as this study with a mean age of 38.3 years and 63% of users being women [6]. Furthermore, previous studies have shown older age being associated with impaired digital health literacy [10] and dissatisfaction with digital PHC [23]. Meanwhile, elderly patients have also expressed a wish and a need to move forward and learn more about digital solutions. To do so, appropriate support is needed [10]. These studies highlight the importance of further investigation of a possible negative association between old age and utilization of PHC Online and if enough support is provided to older age populations to promote utilization.

Several studies have indicated a lack of association between geographically impaired access to PHC and utilization of digital PHC. In one study, users of digital PHC were not more likely to be located within a healthcare professional shortage area [8]. In another study, digital PHC growth was not associated with primary care physician supply [6]. Furthermore, the association between urban residency and utilization of digital PHC is well established [4–7]. In contrast, one study (limited to the diagnoses of sinusitis and urinary tract infection) found an association between digital PHC utilization and longer travel distance to the physical clinic [24]. This study’s finding is in line with previous studies but there is a need for future studies to include travel distance to a physical clinic and conditions associated with urbanicity in the analysis of the effect of geographical differences on utilization of digital PHC.

The negative association between CNI and utilization of PHC Online seen in this study is in line with results from previous studies that indicated social inequity in access to digital PHC [4, 5] and the social gradient in health could explain the negative association between morbidity and utilization seen in this study.

The main contribution of this study is that we cannot find or prove any association between urbanicity and the utilization of a publicly operated digital PHC service. However, the digital service might increase inequity in access based on social conditions and morbidity. In future studies, other measurements of geographical differences (conditions associated with urbanicity, distance to physical clinic) could be used to further examine population-based utilization of digital PHC.

### Strengths and limitations

This study’s major strength is a large sample size consisting of all public PHCCs in Region Skåne (*n* = 83), 31 of 33 municipalities, 3,653 patients, and 4,482 visits over a time period of more than 10 months. Variations between populations, both geographically and over time, are less likely to have affected the validity of the results. Another strength is the use of the variables CNI and ACG which both have been previously found to correlate to health and healthcare needs in a population [19, 21, 22].

The ecological cross-sectional study design represents a limitation with variations within groups and between individuals and causal relationships not being possible to investigate. Limitations in data not including digital PHC visits to private companies could have affected the associations seen in this study’s results. However, patients are encouraged to use the services provided by the healthcare provider to which they are registered. The variable urbanicity is limited since inhabitants are free to register at any PHCC in Sweden and hence do not have to live in the same municipality in which the PHCC they are registered with exists. Yet, it can be assumed that most inhabitants are registered with a PHCC close to where they live. Data on factors that could further affect access to PHCCs was lacking, such as staffing, opening hours and continuity and quality in healthcare. Limitations in available data made adjustment for sex and age in regression analyses impossible. Since sex and age correlate with utilization of PHC and possibly also with CNI and ACG, this could affect the validity of the associations found in this study. How much of the effect of CNI and ACG found in this study is dependent on age is uncertain.

## Conclusions

Geographical differences based on urbanicity do not seem to affect the utilization of PHC Online. Further studies are needed to clarify a possible association to geographical barriers in access to PHC, specifically accounting for factors associated with urbanicity and distance to physical clinics, and how age and sex affect such an association. Low socioeconomic status and high morbidity were associated with lower utilization of PHC online indicating that PHC Online further increases inequity in access to PHC based on social conditions and morbidity, but further studies on how utilization of PHC Online affects these populations’ utilization of physical PHC are needed. Future studies could also focus on if and how digital PHC can be designed to promote equitable access to PHC and if digital services represent an effective use of resources.

## Data Availability

Data are available from the corresponding author on reasonable request.

## Abbreviations

ACG: Adjusted Clinical Groups
CNI: Care Need Index
Digital PHC: Direct-to-consumer telemedicine service
ICD-10: International Statistical Classification of Diseases and Related Health Problems
PHC: Primary healthcare
PHCC: Primary healthcare center
PHC Online: Primary Healthcare Scania Online
RSVD: Region Skåne’s Health Care Databases
SES: Socioeconomic status
VIF: Variance Inflation Factor
